# Eating as an Automatic Behavior

**Published:** 2007-12-15

**Authors:** Deborah Cohen, Thomas A Farley

**Affiliations:** RAND Corporation; Tulane University School of Public Health and Tropical Medicine, New Orleans, Louisiana

## Abstract

The continued growth of the obesity epidemic at a time when obesity is highly stigmatizing should make us question the assumption that, given the right information and motivation, people can successfully reduce their food intake over the long term. An alternative view is that eating is an automatic behavior over which the environment has more control than do individuals. Automatic behaviors are those that occur without awareness, are initiated without intention, tend to continue without control, and operate efficiently or with little effort.

The concept that eating is an automatic behavior is supported by studies that demonstrate the impact of the environmental context and food presentation on eating. The amount of food eaten is strongly influenced by factors such as portion size, food visibility and salience, and the ease of obtaining food. Moreover, people are often unaware of the amount of food they have eaten or of the environmental influences on their eating. A revised view of eating as an automatic behavior, as opposed to one that humans can self-regulate, has profound implications for our response to the obesity epidemic, suggesting that the focus should be less on nutrition education and more on shaping the food environment.

## Introduction

Perhaps the most puzzling and frustrating aspect of the obesity epidemic is the contrast between our understanding of the biology of the problem and our inability to halt the epidemic. During the past 25 years, the percentage of Americans who are obese (body mass index [BMI] ≥30.0 kg/m^2^) has increased from 14.5% to 32.2% (1,2). Much remains to be learned about the metabolism of macronutrients, but no one has seriously challenged the proposition that obesity results from caloric intake being chronically greater than caloric expenditure. Why people continue to consume more calories than they need when the consequences are so apparent, stigmatizing, and widely understood to be unhealthy is a question that remains unanswered.

The obesity epidemic can only have surged in recent decades because of a decline in physical activity levels, an increase in caloric consumption, or both. Although the contribution of physical inactivity to the problem is still unclear, both surveys of food consumption and data on the quantity of food distributed in the United States suggest that caloric consumption of the average American has increased during this time (3,4). This evidence should focus our attention on the most important question in this epidemic: why do people eat too much?

National efforts to treat and prevent obesity depend to a large degree on educating people to regulate their food intake through such means as publicizing general guidelines on nutrition, promoting tailored diets, and labeling foods with nutrition information (5,6). The continued growth of the epidemic despite the employment of these techniques should make people question the assumptions underlying them. The fundamental assumption is that, given the right information and motivation, people can successfully reduce their food intake to match their caloric expenditure over the long term. This assumption in turn implies that eating is a conscious act. An alternative assumption is that eating is a behavior controlled by the environment rather than by the individual. This idea is supported by research on both the environmental influences on eating and the automatic nature of certain behaviors (7).

## Environmental Influences on Eating

Many studies in recent years have demonstrated the powerful influences of the environment on the amount of food people consume. Food portion sizes in particular appear to be very important to consumption patterns; people served larger portions simply eat more food, regardless of their body weight and regardless of the food item, meal setting, or timing of other meals (8-12). For example, people at a restaurant who were served a baked pasta dish that was 50% larger than the normal portion ate 43% more than people served the normal portion of the dish, increasing their caloric consumption at the meal by 159 kcal (8). Men given 175-g bags of potato chips tripled the amount of chips they ate compared with men given 25-g bags of potato chips, taking in an extra 311 kcal (11). The temptation to eat food at hand is so strong that human beings eat more even if the food tastes bad. In one study, people at a movie theater who were given popcorn in boxes twice the normal size and that was 14 days old complained about the taste but still ate 34% more popcorn than did people given stale popcorn in boxes that were the normal size (13).

Beyond portion size, one principle is that the amount of food consumed increases as the effort to eat it decreases, even if the differences in effort are tiny. For example, office workers who had chocolate Kisses within reach on their desks ate an average of 5.6 more candies (total of 136 kcal) per day than did workers for whom candy was placed on a shelf 2 m away (14). The kilocalorie amounts demonstrated by these experiments (8-14) are significant, because a small caloric imbalance over time can produce obesity. In fact, by one estimate, the median weight gain in the obesity epidemic over the last two decades could be caused by a daily excess of only 100 kcal to 150 kcal (3).

The mere sight of food can stimulate people to eat. For example, Wansink et al showed that office workers ate 3.1 more chocolate Kisses (total of 75 kcal) when the candy was placed on their desks in transparent jars than when the candy was placed in opaque jars (15). In another clever experiment by Wansink et al, researchers secretly refilled bowls of soup while people ate from them and found that people ate 73% more soup when this occurred (16). Wansink's work demonstrates repeatedly that environmental cues influence the frequency and quantity of what people eat and that people do not typically recognize these cues (15-17).

The context in which eating takes place can also greatly influence consumption patterns. The longer the meal, the more people eat (18). The amount of food people eat is directly and strongly related to the number of people sharing the meal, with food consumption increasing by 28% when one other person is present and increasing steadily to 71% when the number of companions is six or more, according to the findings of one study (19). Eating with other people also introduces other powerful social effects. In one study, people given two bowls of crackers, one containing goldfish crackers and one containing animal crackers, were much more likely to eat the type of crackers that a person they were speaking to was eating, without having any awareness that they were copying the other person (20). Moreover, people subsequently reported that they preferred the crackers the other person had eaten, without recognizing that their preference was influenced by the other person.

## Environment-Perception-Behavior Sequence

Researchers in recent decades have made progress unrelated to eating or obesity in understanding how human beings respond to environmental stimuli. The environment is the context in which human beings act and react. Every moment, as people look and listen to what is going on around them, they perceive features of their environments. Some of those perceptions occur without awareness, and many behavioral responses similarly occur without awareness or conscious thought.

Psychologists have shown that behavioral responses to environmental stimuli, whether the behaviors appear to be instinctive or deliberate, can be influenced by what has been termed *priming*, or the manipulation of decisions and judgments by the previous presentation of words, concepts, or images that are not perceived as being related to the task at hand (7,20-22). The effects of priming can be surprising. For example, Bargh et al primed subjects by having them solve word puzzles containing words related to anger, such as *rude*, *impolite*, and *obnoxious*; afterward, these subjects were more likely to interrupt a conversation than were people primed with neutral words or words related to politeness (23). North and colleagues showed that when they played French music in a wine store people bought more French wines, and when they played German music people bought more German wines, with little or no awareness of the effect of the music on their purchases (24). An example of how food consumption can be influenced by priming was shown in a study in which thirsty subjects shown a "happy face" subsequently drank more of a fruit-flavored drink and rated it more favorably than did subjects shown an "angry face" (25).

Another important determinant of how human beings respond to any feature of their environment is simply its salience, that is, how much it attracts their attention. For example, marketing researchers have shown that when the amount of shelf space for a consumer item is doubled in grocery stores, sales of that item increase by about 40% (26,27). This effect is seen regardless of whether the item is generally popular or unpopular. Sales also increase when special displays and end-aisle displays are used and when items are placed at eye level (27,28). Grocery chains aware of this principle maximize their revenue by arranging large, prominent displays of high-profit items.

## Automatic Behaviors

Human beings have limited cognitive capacities, with the ability to consciously process only 40 to 60 bits of information per second — equivalent to a short sentence. However, their entire processing capacity, which includes the visual system and the unconscious, is estimated to be 11 million bits per second (29). Therefore, the brain needs mechanisms that do not require cognitive awareness to perceive the environment and react to it. Indeed, human beings' ability to be effective, high-functioning beings depends not only on their ability to think abstractly and creatively but also on their ability to free their minds for this higher-level thinking by assigning routine tasks to lower-level brain involvement. Therefore, noncognitive behaviors are not a sign of weakness but rather an adaptation that allows human beings to be a uniquely productive species (7,30).

In recent years, psychologists have developed a greater understanding of the *automatic* behaviors, which can be defined as those that operate without cognitive direction (30,31). A great deal of mental effort is required to make conscious decisions and then implement them in the form of behaviors (30). Most of our responses to our environment can be understood as automatic behaviors. Human beings smile or laugh when amused, frown when annoyed, become startled when surprised by a loud noise, and tense their muscles when threatened, all without making any conscious decision or being aware of the behavior. An example of a more complex automatic behavior is social mimicry. In conversation, people copy others' mannerisms, such as smiling, rubbing their face, and shaking their feet, regardless of whether they are acquainted with the other people and without the slightest recognition that they are copying them (32).

Bargh has defined four characteristics of automatic behaviors: 1) they occur without awareness, 2) they are initiated without intention, 3) they continue once initiated without control, and 4) they operate efficiently or with little effort (33). However, not all of these criteria are required for a behavior to be considered automatic. Studies on food consumption indicate that eating should be viewed as an automatic behavior (8,9,12,14,15,34,35). People are generally not aware of how much they are eating. In studies demonstrating the influence of portion size on eating, people given large portions usually did not believe they had eaten more than people given normal-sized portions, and when surveyed afterwards, they did not report greater feelings of fullness compared with people eating smaller portions (8,9,12,34). Evidence that eating begins without conscious intent can be taken from both the tendency to eat any food that is in sight or at arm's length (14,15,35) as well as the finding that people are more likely to eat simply because it is mealtime than because they are hungry (36). Once people initiate eating, they usually continue until the food is gone or until some other external occurrence changes the situation. In one study, people were less likely to stop eating because they were full than because no food or drink remained, they had no time to eat more, or they had finished watching television (36). These studies also demonstrate that the natural trajectory of eating — that is, what takes place without conscious effort — is for it to continue. Effort is not required to continue eating when food is present; effort is required to refrain from eating when food is present.

It is intuitive that behaviors central to a human being's or animal's survival are automatic. Evaluations of the safety of our surroundings and judgments of the potential danger posed by strangers are often automatic and typically based upon stereotypes (23,37). The "fight or flight" response would be too slow to be protective if it required deliberate decision-making. Because a central evolutionary task of human beings has been to consume enough energy to live, it is not surprising that we are programmed to eat whenever food is within reach.

Characterizing eating as an automatic behavior does not mean that human beings cannot bring eating under volitional control. People certainly can refuse dessert or resist the temptation of the chocolates in the jar on the desk. All automatic behaviors can be controlled temporarily. Human beings can consciously prevent themselves from smiling when amused, frowning when annoyed, or tensing their muscles when threatened. It just takes effort. But the amount of effort required to refrain from eating when food is present is substantial, and it is nearly impossible to sustain over the long term. For example, in a study on self-control, Baumeister and colleagues allowed members of one study group to eat freshly baked chocolate-chip cookies while members of a second group given access to the cookies were told to refuse them and were allowed to eat only radishes; members of a third control group had no food to eat or to refuse (38). Afterward, the researchers asked the members of the three groups to work on an unsolvable puzzle. Members of the control group given no food worked on the puzzle for 21 minutes before quitting, and members of the group that were allowed to eat the cookies worked for 19 minutes. In sharp contrast, members of the group that had to refuse the cookies quit after only 8 minutes, and they reported more fatigue than members of the other groups. The work of refusing tempting food required mental effort — enough to deplete participants' ability to perform other higher-level processes.

In general, human self-control over automatic behaviors is limited. Self-control tires like a muscle and taxes our ability to perform other tasks (39). And just as refusing food depletes a person's mental reserves, tasks requiring mental effort can reduce the ability to resist the temptation of food. In one study, people trying to maintain a diet who were deliberately frustrated with an unsolvable problem increased their food intake compared with people who were not trying to control their eating (40). The high mental demands of dieting may partially explain the commonly observed pattern of dieters initially losing weight and then gaining it back (41).

Automatic behaviors share another important characteristic. Because people are unaware of the behaviors, they are also unaware that the behaviors are not under their control. Nisbett and Wilson have shown that people are often unaware of a particular stimulus that elicits a response, and even if they are aware of both the stimulus and the response, they may be unaware that the stimulus actually caused the response. Instead, people tend to fabricate reasons to explain their behaviors, typically choosing the most plausible, culturally acceptable theories (42). Bargh and Chartrand found that, even after people have been shown the results of experiments demonstrating the automatic nature of their actions, they steadfastly refuse to believe that those actions did not result from conscious choice (30). Our difficulty as a society in accepting how strongly our environment influences eating may stem from our inability to recognize and our refusal to accept our own eating as an automatic behavior. We blame our lack of willpower on the inability to maintain a diet, when it is more likely that our automatic responses to ubiquitous cues to eat and the availability of cheap, convenient, calorie-dense food are responsible.

If the behavior of eating were automatic, one would predict that it would favor foods that are most available and most visible and that require the least effort to eat — such as precooked and prepackaged foods and beverages that can be eaten without utensils. In fact, the foods that have shown the greatest increase in sales in the past quarter century meet this description: soft drinks, salty snacks, French fries, and pizza (43).

## Comment

Assuming that people who are overweight are simply unconcerned about their weight is tempting. But most Americans consider themselves to be overweight, and nearly one-third are actively trying to lose weight (including nearly one-fourth of women of normal weight [BMI = 18.5–25.0 kg/m^2^]) (44,45). The observation that so many people continue to gain weight despite wanting to be thin is more accurately explained by describing eating as an automatic behavior.

A revised view of eating as an automatic behavior, as opposed to a behavior that human beings can self-regulate, has profound implications for our response to the obesity epidemic. Indeed, researchers have described high levels of food marketing, accessibility, and quantity as the "toxic environment" at the root of the obesity epidemic (46-49). This concept suggests that educational or motivational approaches to reducing population-level consumption, such as the food guide pyramid, nutrition labeling, and dietary counseling, will continue to fail. In place of these approaches, to reduce consumption we should decrease the accessibility, visibility, or quantities of foods to which people are exposed and reduce the cues in our environment that encourage eating. The best approaches include reducing portion sizes, limiting access to ready-to-eat foods, limiting the availability of snack foods in schools and workplaces, and reducing food advertising. Because human beings appear to be very sensitive to small changes in the food environment, these modifications may not need to be large to be effective. Furthermore, because of the automatic nature of eating and because people are currently consuming more calories than they need, these changes — once implemented — might hardly be noticed. This perspective represents our best hope for controlling the obesity epidemic.
